# Screening and Evaluation of Host-Derived Intestinal Probiotic Candidates *Lactococcus* sp. and *Bacillus* sp. from Largemouth Bass for Bacterial Disease Biocontrol

**DOI:** 10.3390/microorganisms14102343

**Published:** 2026-10-14

**Authors:** Jie Yu, Qun Lu, Qian-Qian Ma, Hui-Hui Hu, Jie Leng, Cheng-Ke Zhu, Ming-Ji Lv, Song Zhu

**Affiliations:** 1 College of Fisheries, Southwest University, Chongqing 400715, China; 2 Chongqing Institute of Fisheries Sciences, Chongqing 400020, China; 3 Key Laboratory of Freshwater Fish Reproduction and Development (Ministry of Education), Key Laboratory of Aquatic Science of Chongqing, Southwest University, Chongqing 400715, China

**Keywords:** *Micropterus salmoides*, host-derived probiotics, antagonistic activity, protective efficacy

## Abstract

Bacterial infections represent a major constraint to largemouth bass (*Micropterus salmoides*) aquaculture; safe and sustainable alternatives to conventional antibiotics are needed. This study aimed to screen, characterize, and evaluate host-derived intestinal probiotics from healthy largemouth bass for bacterial disease biocontrol. A total of 68 intestinal bacterial strains were isolated and screened for antagonistic activity against 21 bacterial pathogens previously isolated from diseased largemouth bass. Five isolates with stable inhibitory activity were identified as *Plesiomonas shigelloides* (MS-24), *Aeromonas dhakensis* (MS-26), *Lactococcus lactis* (MS-54), *Bacillus subtilis* (MS-67), and *Bacillus paranthracis* (MS-68) by *16S rRNA* and *gyrB* gene sequencing. Morphological characteristics, physiological and biochemical profiles, and growth kinetics of the 5 isolates were assessed. Among them, only MS-54 and MS-67 exhibited non-hemolytic phenotypes and low in vivo toxicity, with host mortality rates of 0.00% and 1.11%, respectively. In challenge trials, dietary supplementation with MS-54, MS-67, or their combination reduced cumulative mortality to varying degrees. Significant protection was observed against *V. alginolyticus* and *A. jandaei*, and the highest relative percent survival (RPS) was 22.62% in the MS-54 + MS-67 group after *A. jandaei* challenge. Results so far indicated that *Lactococcus lactis* MS-54 and *Bacillus subtilis* MS-67 are promising host-derived probiotic candidates with good antagonistic activities, providing potential strategies for bacterial disease biocontrol and sustainable management in largemouth bass aquaculture.

## 1. Introduction

Largemouth bass (*Micropterus salmoides*) has become an important freshwater aquaculture species worldwide owing to its rapid growth, strong environmental adaptability, and high nutritional value [1]. However, the rapid intensification of largemouth bass farming has been accompanied by frequent outbreaks of infectious diseases, particularly those caused by bacterial pathogens such as *Aeromonas* spp. and *Pseudomonas* spp. [2,3]. In our previous epidemiological surveillance across 12 largemouth bass farming regions, 21 putative bacterial pathogens were isolated from diseased fish. These pathogens were associated with severe clinical manifestations, including ulceration, enteritis, ascites, and mass mortality, resulting in substantial economic losses [4].

Disease control in aquaculture has historically relied on broad-spectrum antibiotics and chemotherapeutic agents [5]. Although these treatments can be effective in the short term, their extensive and sometimes inappropriate use has raised serious ecological and public health concerns, including the emergence and dissemination of antimicrobial resistance and the accumulation of chemical residues in aquatic environments and aquaculture products [6,7]. Given the diversity of bacterial pathogens in largemouth bass and the limitations of current disease-control strategies, there is an urgent need to develop safe, environmentally friendly, and sustainable alternatives to conventional antibiotics for disease prevention and control.

Probiotics, defined as live microorganisms that confer benefits to the host, have attracted increasing attention as promising tools for disease prevention and control [8,9,10]. Among probiotic candidates, host-derived strains isolated from the gastrointestinal tract of healthy hosts may have advantages over allochthonous strains, as they are likely to be better adapted to the host intestinal environment and may exhibit greater colonization ability, ecological compatibility, and persistence within the gut ecosystem [11,12]. Therefore, the intestinal microbiota of healthy largemouth bass represents a valuable reservoir for screening host-adapted probiotic candidates with potential applications in species-specific disease control. Recent studies have reported the isolation and identification of probiotic candidates from the intestinal tract of largemouth bass for the prevention of bacterial infections [13,14]. Nevertheless, systematic screening of cultivable host-derived intestinal bacteria with antagonistic activity against diverse pathogens remains limited.

Therefore, the present study aimed to isolate cultivable intestinal bacteria from healthy largemouth bass and screen them against 21 bacterial pathogens previously isolated from diseased largemouth bass. Probiotic isolates were identified using *16S rRNA* and *gyrB* gene sequencing and further characterized in terms of morphology, biochemical properties, growth performance, and biosafety. Two candidate strains, *Lactococcus lactis* MS-54 and *Bacillus subtilis* MS-67, were subsequently administered to largemouth bass either individually or in combination, and the protective efficacy was evaluated through experimental bacterial challenge trials. This work provides candidate host-derived probiotic strains for the development of antibiotic-alternative and species-specific bacterial disease control strategies for largemouth bass aquaculture.

## 2. Materials and Methods

### 2.1. Bacterial Strains, Fish, and Ethical Statement

A total of 21 bacterial pathogens previously isolated from diseased largemouth bass and preserved at −80 °C were used as indicator strains. These strains included *Aeromonas caviae*, *Aeromonas dhakensis*, *Aeromonas hydrophila*, *Aeromonas jandaei*, *Aeromonas salmonicida*, *Aeromonas veronii*, *Chryseobacterium indologenes*, *Edwardsiella piscicida*, *Enterococcus faecalis*, *Lactococcus garvieae*, *Photobacterium damselae*, *Plesiomonas shigelloides*, *Proteus vulgaris*, *Pseudomonas aeruginosa*, *Pseudomonas parafulva*, *Pseudomonas putida*, *Shewanella xiamenensis*, *Streptococcus agalactiae*, *Vibrio alginolyticus*, *Vibrio cholerae*, and *Vibrio parahaemolyticus* (Tu et al., 2025 [4]).

Largemouth bass used for candidate antagonistic bacteria isolation, biosafety and protective efficacy tests were obtained from Chongqing Xiaoxiao Agricultural Co., Ltd. (Chongqing, China). Before sampling, fish were acclimated for two weeks in glass aquaria under controlled conditions, with a water temperature of approximately 28 °C and dissolved oxygen above 5 mg/L. Fish were fed a commercial diet twice daily at 09:00 and 17:00 at apparent satiation, and one-third of the water was renewed daily. All experimental procedures involving animals strictly complied with guidelines approved by the Ethics Review Committee for Laboratory Animals at Southwest University (Approval No.: SWU-AF-2024-003).

### 2.2. Isolation of Cultivable Intestinal Bacteria

Largemouth bass (*n* = 10; body length: 5.91 ± 0.42 cm; body weight: 2.54 ± 0.25 g) were surface-disinfected with 75% ethanol, and the intestinal tract was aseptically excised. The intestinal contents were gently removed using sterile forceps. The remaining intestinal tissue was finely minced, transferred into a centrifuge tube containing 1 mL of sterile physiological saline, and thoroughly homogenized with a tissue homogenizer (Thunderscience Co., Ltd., Shanghai, China). The homogenate was serially ten-fold diluted with sterile physiological saline to 10^−5^. Aliquots of 0.1 mL from the 10^−3^, 10^−4^, and 10^−5^ dilutions were spread-plated in triplicate onto Luria–Bertani (LB) agar. A plate spread with 0.1 mL of sterile saline was used as the negative control. After the liquid was absorbed for 5 min, the plates were inverted and incubated in a biochemical incubator (Shidukai Instrument and Equipment Co., Ltd., Wuxi, China) at 30 °C for 24–48 h. Single colonies with distinct morphological characteristics, including colony shape, size, and color, were selected and purified by two consecutive subcultures on fresh LB agar. Purified isolates were cryopreserved at −80 °C for subsequent analyses.

### 2.3. Determination of the Antagonistic Activity

The antagonistic activity of the intestinal isolates against the pathogenic indicator strains was evaluated using the agar well diffusion assay. Indicator pathogens and candidate isolates were cultured in LB broth. Bacterial cells were harvested by centrifugation at 6000 rpm for 10 min, and the supernatants were discarded. The cell pellets were resuspended in sterile physiological saline (OD_600_ = 0.6). Aliquots of 100 µL of each pathogen suspension were evenly spread onto LB agar plates. Wells of uniform diameter (6 mm) were aseptically punched into the agar medium. Subsequently, 20 µL of each candidate bacterial suspension was added to the wells, and an equal volume of sterile saline was used as the negative control. The antagonistic activity assay was performed in three independent biological replicates. For each replicate, candidate strains and pathogenic indicator strains were freshly cultured, and the assay was conducted independently. Within each biological replicate, three technical replicate wells were included. The inhibition zones from the three technical wells were averaged within each biological replicate. The final inhibition zone values are presented as the mean ± SD of the three independent biological replicates.

After incubation at 30 °C for 24 h, inhibition zones surrounding the wells were recorded as evidence of antibacterial activity. The inhibition zone was defined as the clear zone without visible growth of the indicator bacterium around the well. For irregular zones, two perpendicular diameters were measured and averaged. When scattered bacterial growth occurred within or near the inhibition area, only the completely clear region was measured. Ambiguous zones without a clearly defined margin were recorded as no detectable inhibition. The diameters of the inhibition zones were measured, including the 6 mm well diameter, in millimeters using a vernier caliper. Isolates showing prominent antagonistic activity in the primary screening were subjected to secondary validation, and those with reproducible inhibitory activity were selected for further characterization.

### 2.4. Molecular Identification of the Probiotic Candidates

Probiotic candidates were inoculated into LB broth and incubated at 28 °C with shaking. When OD_600_ = 0.6, bacterial cells were collected by centrifugation at 4000 rpm for 10 min. Genomic DNA was extracted using a Bacterial Genomic DNA Extraction Kit (Tiangen Biochemical Technology Co., Ltd., Beijing, China) according to the manufacturer’s instructions. The *16S rRNA* and *gyrB* genes were amplified by polymerase chain reaction (PCR) using 2× Rapid Taq Master Mix (Vazyme Biotechnology Co., Ltd., Nanjing, China). The PCR primers and amplification conditions referred to our previous study (Tu et al., 2025 [4]). The PCR products were purified and sequenced by TsingKe Biotechnology Co., Ltd. (Wuhan, China). The obtained sequences were compared with sequences in the National Center for Biotechnology Information (NCBI) database using the Basic Local Alignment Search Tool (BLAST). Phylogenetic trees were constructed using the neighbor-joining method in MEGA 11, and branch support was evaluated based on 1000 bootstrap replicates.

### 2.5. Transmission Electron Microscopy (TEM) Observation

The identified probiotic candidates were inoculated into LB broth and incubated at 28 °C until OD_600_ = 0.6. Bacterial cells were collected by centrifugation at 4000 rpm for 10 min and washed twice with sterile phosphate-buffered saline (PBS) to remove residual medium components. A small aliquot of the resuspended bacterial suspension was applied onto Formvar-coated copper grids. After a brief sedimentation period, the samples were negatively stained with phosphotungstic acid solution. Cell morphology and flagellar arrangement were observed and photographed using a JEM-1400 TEM (JEOL Ltd., Tokyo, Japan).

### 2.6. Physiological and Biochemical Identification

The physiological and biochemical profiles of the probiotic candidates were determined using commercial micro-identification kits (HaiBo Biotechnology Co., Ltd., Qingdao, China) according to the manufacturer’s instructions. The detection indicators include Methyl Red and Voges–Proskauer (MR-VP), o-nitrophenyl-β-D-galactopyranoside (ONPG), urease (Ur), gelatin hydrolysis (GH), arginine dihydrolase (AD), ornithine decarboxylase (OD), lysin decarboxylase (LD), and oxidase (Ox). Halotolerance was assessed by cultivating the strain in tryptone water supplemented with varying NaCl concentrations (0%, 3%, 6%, 8%, and 10%). Carbohydrate utilization was evaluated in a medium containing 1% NaCl, including the fermentation of mannitol (Mal), mannose (Mae), lactose (La), cellobiose (Ce), arabinose (Ar), and sucrose (Su).

### 2.7. Growth Curve Determination of the Probiotic Candidates

The growth kinetics of the probiotic candidates were evaluated. Purified cultures were inoculated into LB broth and incubated at 30 °C with shaking at 180 rpm. To monitor bacterial growth, 200 µL aliquots were collected at 0, 0.5, 1, 2, 4, 6, 8, 10, 12, 16, 20, 24, 28, and 32 h. The OD_600_ was measured using a microplate spectrophotometer (Shanghai Metash Instruments Co., Ltd., Shanghai, China), with sterile LB broth used as the blank control. All spectrophotometric measurements were performed in triplicate in 96-well microtiter plates. Growth curves were constructed by plotting the mean OD_600_ values against incubation time.

### 2.8. Safety Assessment of the Probiotic Candidates

#### 2.8.1. In Vitro Hemolytic Assay

Hemolytic activity was evaluated by spotting 2 μL aliquots of bacterial suspension (OD_600_ = 0.6) onto sheep blood agar plates. After complete absorption, the plates were inverted and incubated. Hemolytic activity was classified according to the appearance of zones around the colonies. A greenish zone was interpreted as incomplete hemolysis (α-hemolysis). A clear transparent zone was interpreted as complete erythrocyte lysis (β-hemolysis). The absence of a hemolytic zone was interpreted as no hemolytic activity (γ-hemolysis).

#### 2.8.2. In Vivo Biosafety Test

The biosafety of the probiotic candidates was evaluated in healthy largemouth bass. After acclimation, fish were randomly allocated into six groups (30 fish per group, three replicates). The probiotic candidates were cultured in LB broth at 30 °C. Bacterial cells were harvested by centrifugation at 6000 rpm for 10 min, and the resulting pellets were resuspended in sterile PBS to a final concentration of 1 × 10^6^ CFU/mL. Experimental fish were intraperitoneally injected with 50 µL of bacterial suspension (5 × 10^4^ CFU/fish), whereas the control fish were injected with an equal volume of sterile PBS. During the 14-day observation period, fish were fed a basal diet, and clinical signs and mortality were recorded daily.

### 2.9. Protective Efficacy Assessment

To evaluate the protective efficacy of *Lactococcus lactis* MS-54 and *Bacillus subtilis* MS-67 strains, a 30-day feeding trial was conducted. *Lactococcus lactis* MS-54 and *Bacillus subtilis* MS-67 strains were, respectively, cultured in LB broth at 30 °C with agitation at 180 rpm. Cells were harvested by centrifugation, washed twice with PBS, and resuspended to a final concentration of 1 × 10^9^ CFU/mL. Three suspensions were prepared: only the Lactococcus lactis MS-54 strain, only the *Bacillus subtilis* MS-67 strain, and a mixture of *Lactococcus lactis* MS-54 and *Bacillus subtilis* MS-67 strains in a ratio of 1:1. The suspensions were sprayed onto the commercial feed at a dosage of 10 mL per 100 g of feed, while the control diet was treated with an identical volume of sterile PBS. All diets were air-dried in a laminar flow cabinet and prepared daily before use. For the 30-day dietary administration period, fish were randomly allocated into four dietary groups: control, MS-54, MS-67, and MS-54 + MS-67. Each dietary group was maintained in one tank during this period. The tank volume was 5 m^3^, with a working water volume of 3 m^3^, and the stocking density was approximately 200 fish/m^3^. Fish were fed twice daily (09:00 and 17:00).

Following the feeding trial, fish from each dietary group were randomly redistributed for the bacterial challenge experiment. Different challenge treatment groups were established from each dietary group, with three independent replicate tanks per treatment and 30 fish per replicate tank. The pathogenic indicator bacteria were cultured in 10 mL of LB broth at 30 °C. Bacterial cells were harvested via centrifugation, and the resulting pellets were resuspended in sterile PBS to a final concentration of 1 × 10^8^ CFU/mL. Fish in the challenge groups were intraperitoneally injected with 50 μL of the bacterial suspension (5 × 10^6^ CFU/fish), while the negative control group was mock-infected with an equal volume of sterile PBS. Clinical signs and mortality were continuously monitored for 14 days. During the 30-day feeding trial and challenge experiments, water temperature was maintained at 28 °C, dissolved oxygen above 5 mg/L, and pH at 7.8 ± 0.4. The photoperiod was 16 h light:8 h dark. Approximately 25% of the water was renewed daily. To evaluate the protective efficacy, the RPS was calculated as follows:

$$\mathrm{RPS}\left(\%\right)=\left(1-\frac{\mathrm{Mortality}\;\mathrm{rate}\;\mathrm{in}\;\mathrm{the}\;\mathrm{experimental}\;\mathrm{group}}{\mathrm{Mortality}\;\mathrm{rate}\;\mathrm{in}\;\mathrm{the}\;\mathrm{control}\;\mathrm{group}}\right)\times 100$$

### 2.10. Statistical Analysis

Initial data processing relied on Microsoft Excel 2021, and the data for the inhibition zone diameters and the mortality rates of largemouth bass are presented as means ± standard deviation (SD). Cumulative mortality was calculated separately for each challenge tank, and RPS was calculated based on the corresponding mortality values. All statistical analyses were performed using IBM SPSS Statistics 27. Data normality and homogeneity of variances were assessed using the Shapiro–Wilk test and Levene’s test, respectively. When assumptions of normality and homogeneity were satisfied, one-way ANOVA followed by Tukey’s multiple-comparison test was used. When homogeneity of variances was not satisfied, Welch’s ANOVA followed by the Games–Howell test was used. When data did not meet the assumptions of parametric analysis, the Kruskal–Wallis test followed by Dunn–Bonferroni multiple comparisons was applied. Statistical significance was defined at *p* ≤ 0.05, and high significance at *p* ≤ 0.01.

For the protective-efficacy trial, each dietary treatment was administered in a single feeding tank before pathogen challenge. After the feeding period, fish from each dietary-treatment tank were redistributed into three challenge tanks. Therefore, the three challenge tanks represented replication of the challenge condition but not independent replication of the preceding dietary treatment. Accordingly, the mortality and RPS data were analyzed and presented descriptively rather than as independent replicated treatment comparisons.

## 3. Results

### 3.1. Isolation of Cultivable Intestinal Bacteria and In Vitro Antagonistic Activity Assessment

A total of 68 cultivable bacterial strains, designated MS-1 to MS-68, were isolated from the intestinal mucosa of healthy largemouth bass. After primary and secondary antagonistic screening, five candidate strains (MS-24, MS-26, MS-54, MS-67, and MS-68) were selected based on their marked and reproducible inhibitory activity against the tested pathogens.

**Figure 1:**
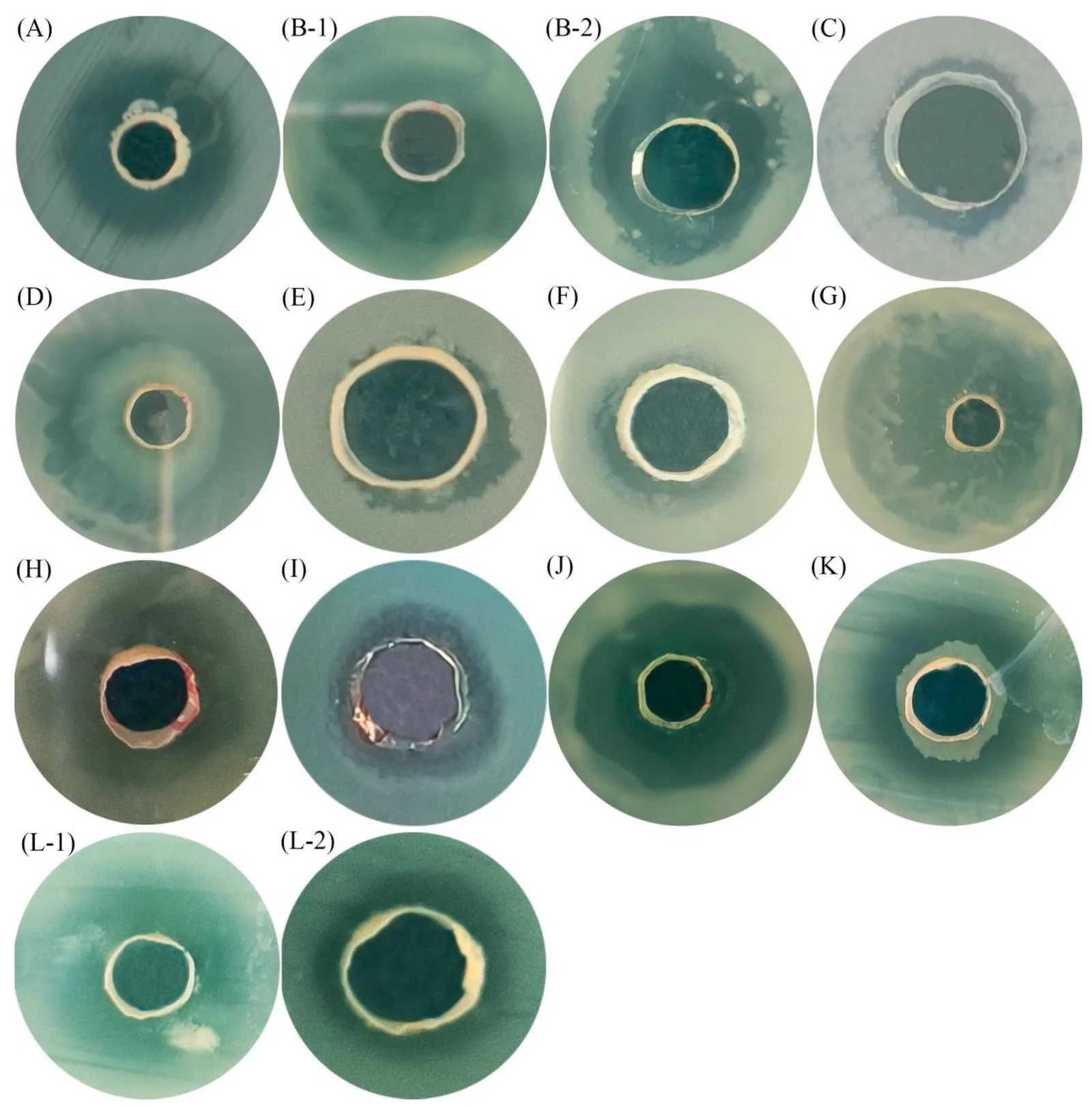
Representative images of the antagonistic activity of the five isolates showing high antagonistic activity. (**A**) MS-67 against *A. jandaei*; (**B-1**) MS-24 against *A. salmonicida*; (**B-2**) MS-54 against *A. salmonicida*; (**C**) MS-54 against *C. indologenes*; (**D**) MS-26 against *E. piscicida*; (**E**) MS-26 against *E. faecalis*; (**F**) MS-24 against *P. damselae*; (**G**) MS-26 against *P. shigelloides*; (**H**) MS-24 against *P. vulgaris*; (**I**) MS-67 against *P. aeruginosa*; (**J**) MS-24 against *P. parafulva*; (**K**) MS-67 against *S. xiamenensis*; (**L-1**) MS-54 against *V. alginolyticus*; (**L-2**) MS-68 against *V. alginolyticus*.

As shown in Figure 1 and Table 1, the five candidate strains inhibited at least one indicator pathogen. Strain MS-68 showed the narrowest inhibitory spectrum and inhibited only *V. alginolyticus*, with an inhibition-zone diameter of 9.2 ± 0.2 mm. In contrast, strains MS-24, MS-26, MS-54, and MS-67 showed broader inhibitory spectra, with inhibition zone diameters ranging from 7.5 ± 0.5 mm to 25.5 ± 0.6 mm. Specifically, the largest inhibition zone was observed for MS-67 against *A. jandaei* at 25.5 ± 0.6 mm. Moreover, MS-26 showed strong antagonistic activity to *Edwardsiella piscicida* and *Plesiomonas shigelloides*, with inhibition-zone diameters of 21.8 ± 0.6 mm and 22.8 ± 1.6 mm, respectively. No antagonistic activity was observed against nine of the tested pathogens, including *A. caviae*, *A. dhakensis*, *A. hydrophila*, *A. veronii*, *L. garvieae*, *P. putida*, *S. agalactiae*, *V. cholerae*, and *V. parahaemolyticus*.

### 3.2. Molecular Identification of the Antagonistic Strains

The *16S rRNA* and *gyrB* genes of the five selected antagonistic isolates were amplified and sequenced to determine their taxonomic affiliations. BLAST analysis showed that all five isolates shared high sequence identity (>99%) with reference strains in the NCBI GenBank database. Phylogenetic analyses based on *16S rRNA* and *gyrB* sequences identified MS-24 as *Plesiomonas shigelloides* (Figure S1), MS-26 as *Aeromonas dhakensis* (Figure S2), MS-54 as *Lactococcus lactis* (Figure S3), MS-67 as *Bacillus subtilis* (Figure S4), and MS-68 as *Bacillus paranthracis* (Figure S5).

### 3.3. TEM Observation

**Figure 2:**
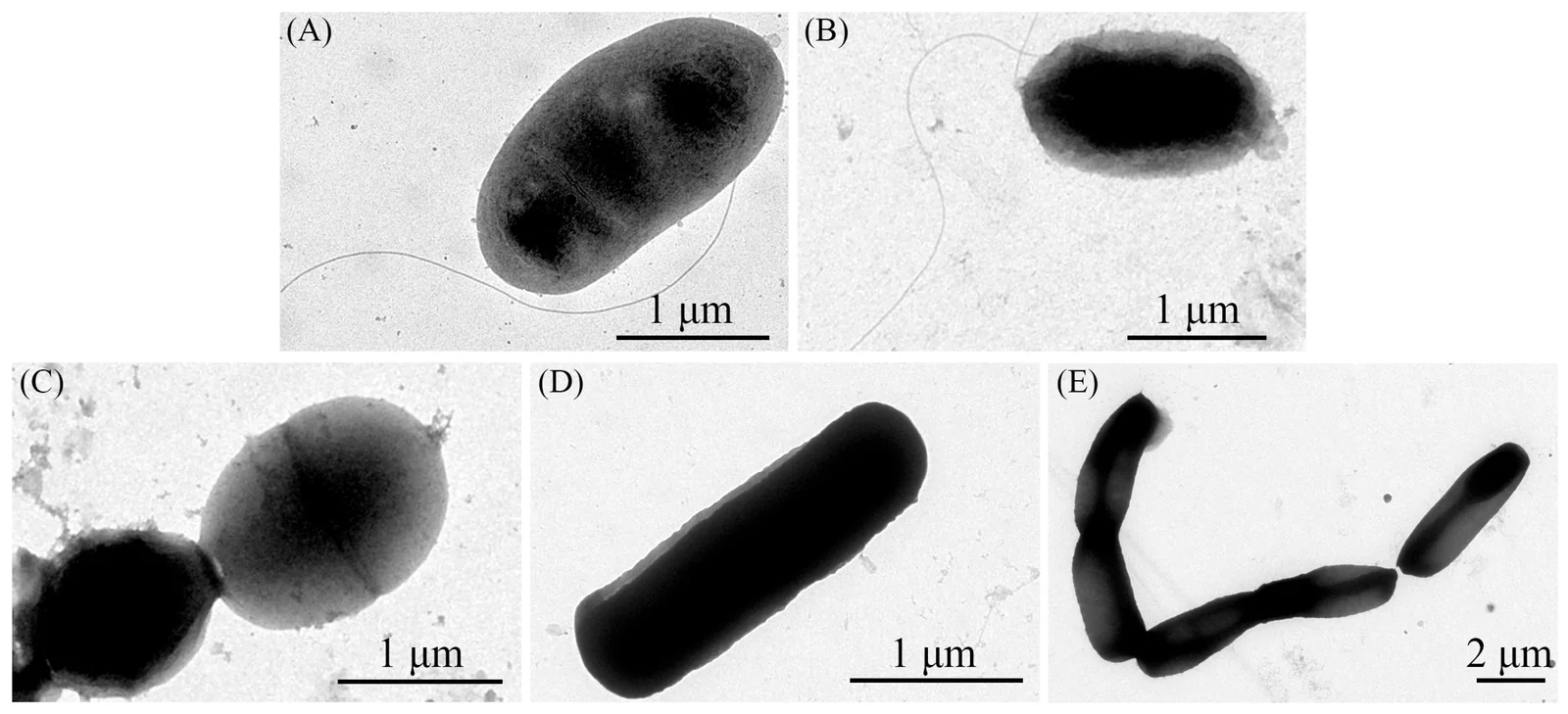
Representative TEM images of the 5 antagonistic strains. (**A**) *Plesiomonas shigelloides* MS-24 (5000×); (**B**) *Aeromonas dhakensis* MS-26 (5000×); (**C**) *Lactococcus lactis* MS-54 (5000×); (**D**) *Bacillus subtilis* MS-67 (5000×); (**E**) *Bacillus paranthracis* MS-68 (2000×).

TEM revealed distinct ultrastructural diversity among the five antagonistic isolates (Figure 2). *Plesiomonas shigelloides* MS-24, *Aeromonas dhakensis* MS-26, *Bacillus subtilis* MS-67, and *Bacillus paranthracis* MS-68 displayed rod-shaped morphology, whereas *Lactococcus lactis* MS-54 showed spherical-to-ovoid morphology. Polar flagella were observed in strains *Plesiomonas shigelloides* MS-24 (Figure 2A) and *Aeromonas dhakensis* MS-26 (Figure 2B). Both *Lactococcus lactis* MS-54 (Figure 2C) and *Bacillus paranthracis* MS-68 (Figure 2E) showed chain-like arrangements. The rod-shaped isolates exhibited notable dimension polymorphism. *Plesiomonas shigelloides* MS-24 and *Aeromonas dhakensis* MS-26 showed short and stout rod-shaped cells, while *Bacillus subtilis* MS-67 (Figure 2D) and *Bacillus paranthracis* MS-68 appeared as elongated rod-shaped cells.

### 3.4. Physiological and Biochemical Characteristics

The physiological and biochemical profiles of the five antagonistic strains are summarized in Table 2. These strains exhibited distinct nutrient utilization profiles. All five isolates grew in 0% and 3% NaCl tryptone water, whereas none grew in 10% NaCl tryptone water. None of the isolates was positive for urease activity. *Plesiomonas shigelloides* MS-24, *Aeromonas dhakensis* MS-26, and *Lactococcus lactis* MS-54 were positive for lactose utilization, whereas *Bacillus subtilis* MS-67 and *Bacillus paranthracis* MS-68 were negative. *Aeromonas dhakensis* MS-26 showed the broadest substrate-utilization profile among the five isolates and was the only strain positive for mannitol and arabinose utilization.

The five antagonistic strains differed in several biochemical reactions. *Aeromonas dhakensis* MS-26, *Bacillus subtilis* MS-67, and *Bacillus paranthracis* MS-68 were positive for MR-VP, whereas *Plesiomonas shigelloides* MS-24 and *Lactococcus lactis* MS-54 were negative. ONPG activity was detected in *Plesiomonas shigelloides* MS-24, *Aeromonas dhakensis* MS-26, and *Bacillus subtilis* MS-67, but not in *Lactococcus lactis* MS-54 or *Bacillus paranthracis* MS-68. Gelatin hydrolysis was positive in *Aeromonas dhakensis* MS-26, *Bacillus subtilis* MS-67, and *Bacillus paranthracis* MS-68. Arginine dihydrolase activity was detected in *Plesiomonas shigelloides* MS-24, *Aeromonas dhakensis* MS-26, *Lactococcus lactis* MS-54, and *Bacillus paranthracis* MS-68. Ornithine decarboxylase and lysine decarboxylase activities were detected in *Plesiomonas shigelloides* MS-24 and *Aeromonas dhakensis* MS-26 only. Oxidase activity was positive in *Aeromonas dhakensis* MS-26, *Lactococcus lactis* MS-54, and *Bacillus subtilis* MS-67, but negative in *Plesiomonas shigelloides* MS-24 and *Bacillus paranthracis* MS-68. Growth at 42 °C was observed for *Plesiomonas shigelloides* MS-24, *Aeromonas dhakensis* MS-26, and *Bacillus subtilis* MS-67, but not for *Lactococcus lactis* MS-54 or *Bacillus paranthracis* MS-68.

### 3.5. Growth Curve Determination of the Antagonistic Strains

**Figure 3:**
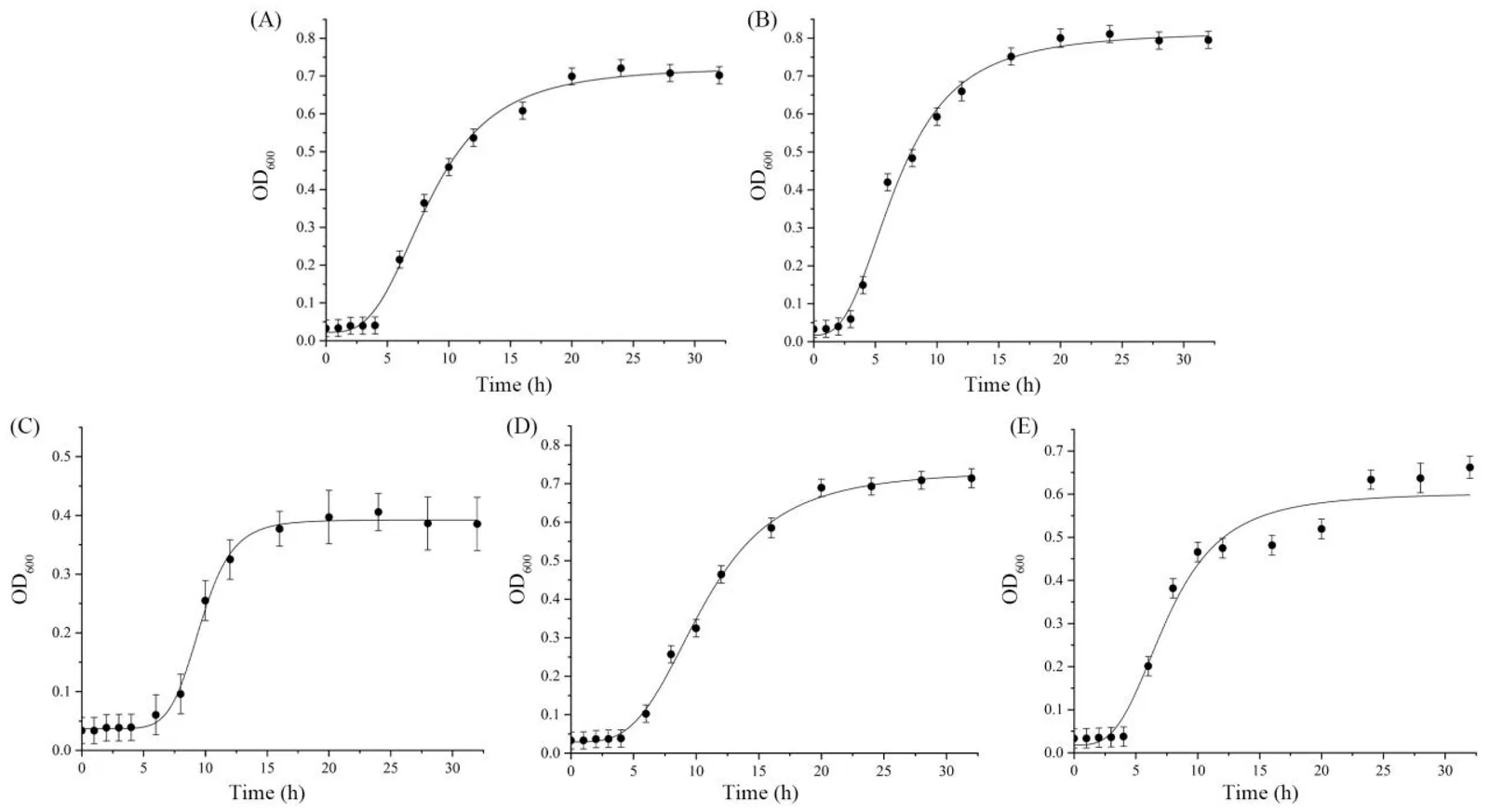
The growth curves of the 5 antagonistic strains. (**A**) *Plesiomonas shigelloides* MS-24; (**B**) *Aeromonas dhakensis* MS-26; (**C**) *Lactococcus lactis* MS-54; (**D**) *Bacillus subtilis* MS-67; (**E**) *Bacillus paranthracis* MS-68.

The growth kinetics of the five selected isolates were evaluated by monitoring OD_600_ over a 32 h incubation period (Figure 3). All five antagonistic strains exhibited a lag phase from 0 to 4 h, followed by exponential growth. *Plesiomonas shigelloides* MS-24, *Aeromonas dhakensis* MS-26, *Bacillus subtilis* MS-67, and *Bacillus paranthracis* MS-68 showed similar growth patterns and entered the stationary phase at approximately 24 h. In contrast, *Lactococcus lactis* MS-54 entered the stationary phase earlier, at approximately 16 h. The maximum optical densities differed among isolates, and *Aeromonas dhakensis* MS-26 reached the highest peak biomass.

### 3.6. Safety Assessment of the Antagonistic Strains

#### 3.6.1. In Vitro Hemolytic Activity

**Figure 4:**
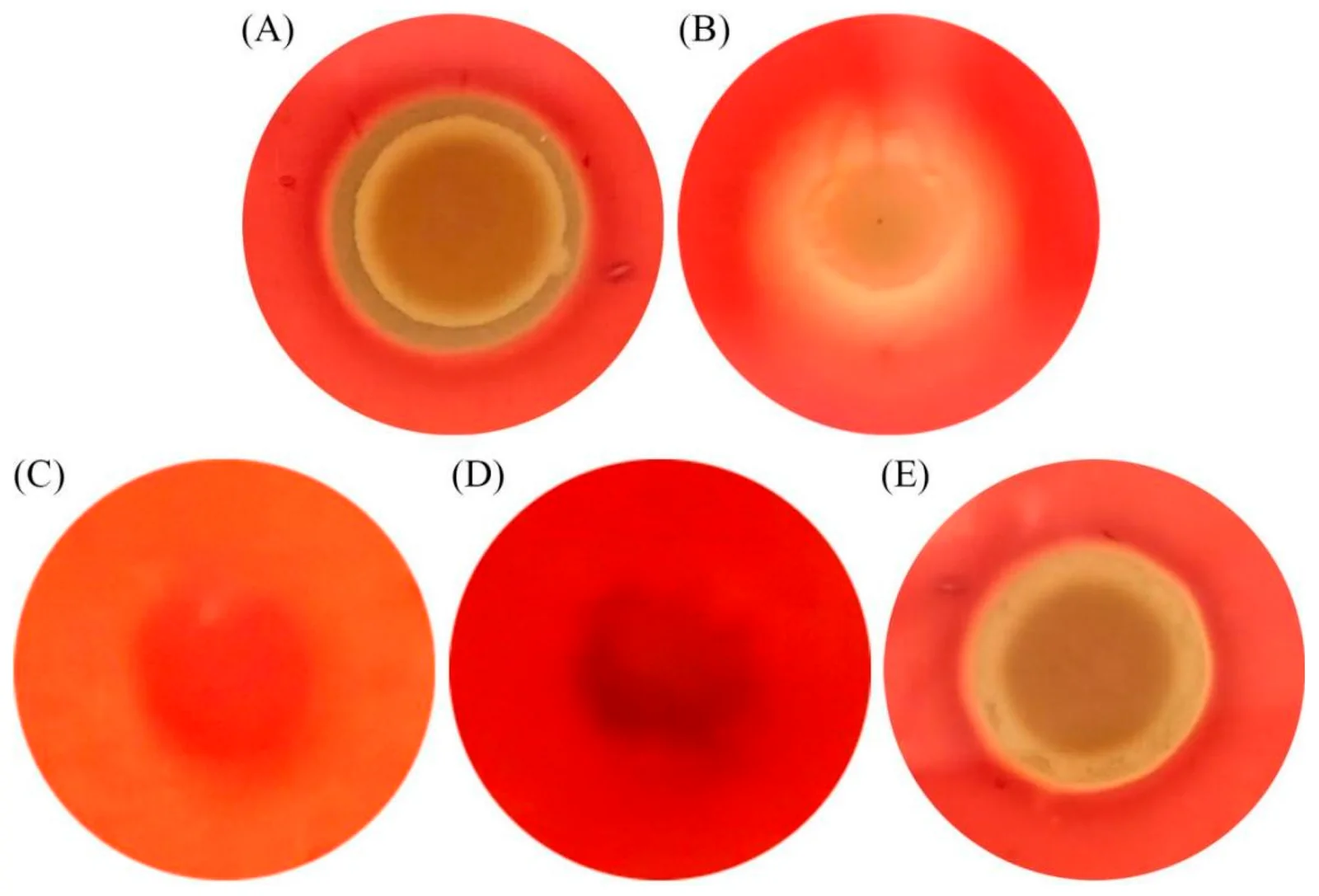
Hemolytic test of the 5 antagonistic strains. (**A**) *Plesiomonas shigelloides* MS-24; (**B**) *Aeromonas dhakensis* MS-26; (**C**) *Lactococcus lactis* MS-54; (**D**) *Bacillus subtilis* MS-67; (**E**) *Bacillus paranthracis* MS-68.

The hemolytic activity of the five antagonistic strains was evaluated on blood agar plates (Figure 4). *Plesiomonas shigelloides* MS-24 showed partial hemolysis and was classified as α-hemolytic. *Aeromonas dhakensis* MS-26 and *Bacillus paranthracis* MS-68 produced clear hemolytic zones and were classified as β-hemolytic. In contrast, *Lactococcus lactis* MS-54 and *Bacillus subtilis* MS-67 showed no visible hemolytic zones and were classified as γ-hemolytic, indicating a non-hemolytic phenotype.

#### 3.6.2. In Vivo Biosafety

The in vivo biosafety of the antagonistic isolates was evaluated in healthy largemouth bass by intraperitoneal injection at 5 × 10^4^ CFU/fish. During the 14-day observation period, high cumulative mortality was recorded in fish injected with *Aeromonas dhakensis* MS-26, *Bacillus paranthracis* MS-68, and *Plesiomonas shigelloides* MS-24, with fatality rates of 96.67 ± 3.33%, 81.11 ± 6.94%, and 67.78 ± 3.85%, respectively (Table 3). In contrast, *Lactococcus lactis* MS-54 and *Bacillus subtilis* MS-67 showed high biosafety under the tested conditions, with fatality rates of 0.00% and 1.11 ± 1.92%, respectively. Based on the non-hemolytic phenotype and low mortality in vivo, *Lactococcus lactis* MS-54 and *Bacillus subtilis* MS-67 were selected for subsequent protective-efficacy evaluation.

### 3.7. Protective Effects of Lactococcus lactis MS-54 and Bacillus subtilis MS-67 on Largemouth Bass

The protective effects of *Lactococcus lactis* MS-54, *Bacillus subtilis* MS-67, and their combination against six bacterial pathogens in largemouth bass are shown in Table 4. Overall, probiotic-treated groups showed lower cumulative mortality than the corresponding pathogen-challenged controls, with RPS values ranging from 5.00% to 22.62%.

For *A. salmonicida*, *C. indologenes*, *P. aeruginosa*, and *S. xiamenensis* challenges, *Lactococcus lactis* MS-54, *Bacillus subtilis* MS-67, or the combined treatment reduced mortality numerically compared with the control groups, but the differences were not significant (*p* > 0.05). Significant reductions in mortality were observed after *V. alginolyticus* and *A. jandaei* challenges (*p* < 0.05). In the *V. alginolyticus* challenge, mortality decreased from 92.22 ± 4.16% in the control group to 81.11 ± 6.85% in the MS-54 group and 80.00 ± 5.44% in the MS-54 + MS-67 group, with RPS values of 12.05% and 13.25%, respectively. In the *A. jandaei* challenge, mortality decreased from 93.33 ± 5.44% in the control group to 73.33 ± 7.20% in the MS-67 group and 72.22 ± 6.28% in the combined group, with RPS values of 21.43% and 22.62%, respectively. These results indicate that *Lactococcus lactis* MS-54 and *Bacillus subtilis* MS-67 provided moderate protection against bacterial challenge, with the clearest protective effects observed against *V. alginolyticus* and *A. jandaei*. However, because each dietary treatment was represented by only one feeding tank before redistribution into challenge tanks, these data are presented as descriptive preliminary findings rather than as statistically independent evidence of dietary-treatment effects.

## 4. Discussion

Bacterial diseases remain a major constraint on the intensive culture of largemouth bass [15,16]. In view of the risks associated with extensive antibiotic use, including antimicrobial resistance and environmental impacts [17,18,19], probiotics have been increasingly considered as alternative or complementary strategies for disease management in aquaculture [20,21,22]. In the present study, 68 cultivable bacterial strains were isolated from the intestinal mucosa of healthy largemouth bass, and five isolates with reproducible antagonistic activity were selected for further evaluation. *Lactococcus lactis* MS-54 and *Bacillus subtilis* MS-67 were, respectively, identified as *L. lactis* and *B. subtilis*, and were retained as probiotic candidates because they combined antagonistic activity and biosafety.

The isolation of antagonistic bacteria from the intestine of healthy fish is consistent with the concept that host-derived microbiota may provide a source of host-adapted probiotic candidates [23]. Host-derived strains are often considered advantageous because they may be better adapted to the physicochemical and ecological conditions of the host gut than allochthonous strains [24,25].

The five selected isolates showed distinct inhibitory spectra against 21 pathogenic bacteria previously associated with diseased largemouth bass [4]. Such variation indicates strain-dependent differences in antagonistic capacity. Comparable strain specificity has been reported for aquaculture probiotics, where inhibitory activity may vary according to the target pathogen, culture conditions, and antimicrobial metabolites produced [26,27]. The use of a broad pathogen panel strengthened the screening relevance of this study, as largemouth bass farms are affected by diverse bacterial agents rather than a single pathogen.

Following validation of the antagonistic capabilities, precise taxonomic identification of these microbial isolates is imperative [28]. Although *16S rRNA* gene sequencing remains a foundational diagnostic tool, supplementary amplification of the *gyrB* gene affords enhanced phylogenetic resolution, particularly for differentiating closely related species within complex genera such as *Bacillus* and *Aeromonas* [29,30]. Employing these robust molecular markers alongside comprehensive phylogenetic analyses, the five highly antagonistic isolates were conclusively identified as *P. shigelloides* (MS-24), *A. dhakensis* (MS-26), *L. lactis* (MS-54), *B. subtilis* (MS-67), and *B. paranthracis* (MS-68). The identification results also emphasized the need for safety screening, as several antagonistic isolates belonged to taxa that may include opportunistic or pathogenic members.

Bacterial morphology is a pivotal evolutionary adaptation that fundamentally influences motility and nutrient acquisition dynamics [31]. The observation of polar flagellar structures in strains *Plesiomonas shigelloides* MS-24 and *Aeromonas dhakensis* MS-26 indicates a specialized adaptation for swimming motility, facilitating rapid chemotactic responses and initial colonization within fluid aquatic environments [32]. The spherical-to-ovoid morphology of *Lactococcus lactis* MS-54 is consistent with classical *Lactococcus* morphotypes [33]. Furthermore, the morphological difference observed between the *Bacillus subtilis* MS-67 and *Bacillus paranthracis* MS-68 likely reflects adaptive autolysin-regulated strategies that confer resistance against protozoan grazing within competitive microenvironments [34,35].

The in vitro growth profiles demonstrated an abbreviated lag phase (0–4 h) across all isolates, denoting rapid acclimation to culture conditions. Notably, *Lactococcus lactis* MS-54 exhibited a highly accelerated exponential phase, achieving a stationary phase within approximately 16 h. Such rapid proliferation facilitates the swift occupation of mucosal niches within the host intestine, thereby preemptively outcompeting slower-growing opportunistic pathogens for essential adhesion sites [20,36].

Antagonistic activity is inextricably linked to the robust carbohydrate metabolic capacities, which supply the necessary bioenergetic substrates for proliferation [37]. Physiological and biochemical characteristics revealed different substrate utilization patterns of the 5 antagonistic strains. These distinct metabolic profiles imply an evolutionary strategy to minimize direct inter-strain resource competition within the complex gut microbiome [38]. Concurrently, environmental resilience, particularly osmotic stress tolerance, is pivotal for ensuring probiotic survivability during feed processing and subsequent gastrointestinal transit [39,40]. While all isolates proliferated under standard salinity (0–3% NaCl), *Aeromonas dhakensis* MS-26 and *Bacillus paranthracis* MS-68 demonstrated exceptional halotolerance, sustaining viability at up to 8% NaCl. The elevated osmotic threshold is likely mediated by adaptive physiological mechanisms, which protect cellular integrity against hypertonic shock [41,42]. Furthermore, all five isolates tested negative for Ur activity. The absence of this enzyme is recorded as a supplementary safety-related trait, confirming that these host-derived strains do not generate ammonia or analogous cytotoxic nitrogenous, which can induce intestinal tissue damage or disrupting the host digestive microenvironment [43,44].

Safety is a prerequisite for probiotic development in aquaculture [45]. In the present study, *Lactococcus lactis* MS-54 and *Bacillus subtilis* MS-67 were non-hemolytic and caused low mortality in largemouth bass after intraperitoneal injection. In contrast, *Plesiomonas shigelloides* MS-24, *Aeromonas dhakensis* MS-26, and *Bacillus paranthracis* MS-68 showed α- or β-hemolysis and caused high mortality, indicating that these isolates are unsuitable for probiotic development despite their antagonistic activity [46,47].

The challenge results showed that *Lactococcus lactis* MS-54 and *Bacillus subtilis* MS-67 provided partial protection against bacterial infection. Significant reductions in mortality were observed only after *V. alginolyticus* and *A. jandaei* challenges. *L. lactis* has been widely studied as a lactic acid bacterium with potential benefits for fish health, including organic acid production, competitive exclusion, bacteriocin production, and immunomodulation [48,49]. *B. subtilis* is also commonly evaluated in aquaculture because of its spore-forming capacity, environmental stability, enzyme production, and ability to produce antimicrobial lipopeptides [50].

Several possible mechanisms may explain the observed antagonistic and protective effects, although these mechanisms were not directly tested in the present study. First, *Lactococcus lactis* MS-54 and *Bacillus subtilis* MS-67 may inhibit pathogens through extracellular antimicrobial metabolites. *L. lactis* strains can produce bacteriocins, while *B. subtilis* strains may synthesize antimicrobial peptides and lipopeptides with activity against Gram-positive and Gram-negative bacteria [51,52]. Second, dietary administration may enhance competitive exclusion by promoting occupation of intestinal niches and competition for nutrients or adhesion sites. Third, probiotics may modulate innate immune responses in fish, including lysozyme activity, complement activity, phagocytosis, antioxidant enzymes, and inflammatory signaling [20,53].

The application of multi-strain formulations frequently yields synergistic protection [54,55]. Although the combined *Lactococcus lactis* MS-54 + *Bacillus subtilis* MS-67 treatment broadened the range of pathogens for which numerical mortality reduction was observed, it did not consistently outperform the corresponding single-strain treatments. The combination produced the highest RPS against *A. jandaei* at 22.62%, but for *C. indologenes* and *S. xiamenensis*, the RPS values of the combined treatment were slightly lower than those of the single-strain treatment. This pattern may reflect differences in strain compatibility, growth dynamics, metabolite production, or effective dose when the two strains are co-administered [55,56]. Future studies should therefore evaluate whether *Lactococcus lactis* MS-54 and *Bacillus subtilis* MS-67 act additively, synergistically, or independently in the fish gut.

The relatively low RPS values may be related to the challenge model. Intraperitoneal injection is a standardized and reproducible method for evaluating disease resistance, but it bypasses important natural defense barriers, including the skin, gills, intestinal mucosa, and mucus layer. Because orally administered probiotics are expected to act mainly at mucosal surfaces, intraperitoneal challenge may underestimate their protective effects in natural infection routes. In immersion, cohabitation, or oral challenge models, probiotics may have greater opportunity to interfere with pathogen adhesion, invasion, and early colonization. Therefore, the present results should be viewed as evidence of partial systemic protection under a stringent challenge condition, rather than a complete assessment of field-level probiotic efficacy.

This study has several limitations: (1) whole-genome sequencing of the antagonistic strains was not performed; (2) colonization and persistence of *Lactococcus lactis* MS-54 and *Bacillus subtilis* MS-67 in the largemouth bass intestine were not measured; (3) although the challenge trial included three independent replicate tanks per treatment, the 30-day probiotic administration period was not replicated at the tank level; (4) the antimicrobial metabolites responsible for the observed inhibition zones were not purified or identified; (5) intestinal bacteria were isolated only on LB agar under aerobic incubation at 30 °C; (6) gut microbiota changes after probiotic administration were not investigated; and (7) the efficacy of *Lactococcus lactis* MS-54 and *Bacillus subtilis* MS-67 remains to be validated under commercial farming conditions.

## 5. Conclusions

In conclusion, 68 cultivable bacterial strains were isolated from the gastrointestinal tracts of healthy largemouth bass. Two strains, MS-54 (*L. lactis*) and MS-67 (*B. subtilis*), were selected as promising host-derived probiotic candidates. Dietary administration of *Lactococcus lactis* MS-54, *Bacillus subtilis* MS-67, or their combination reduced mortality after the related bacterial challenge, with significant protection observed against *A. jandaei* and *V. alginolyticus*. Future research should focus on antagonistic mechanisms, intestinal colonization capacity, and gut microbiota analysis. In addition, dose and strain ratio optimization, feeding duration, and field validation are required before these strains can be recommended for practical application in largemouth bass aquaculture.

## Figures and Tables

**Table 1:** In vitro antagonistic activity of the five isolates against 21 pathogens.

Pathogen	Mean Inhibition Zone Diameter (mm)				
	*P. shigelloides* MS-24	*A. dhakensis* MS-26	*L. lactis* MS-54	*B. subtilis* MS-67	*B. paranthracis* MS-68
*Aeromonas caviae*	-	-	-	-	-
*Aeromonas dhakensis*	-	-	-	-	-
*Aeromonas hydrophila*	-	-	-	-	-
*Aeromonas jandaei*	-	-	-	25.5 ± 0.6	-
*Aeromonas salmonicida*	14.8 ± 0.8	-	12.1 ± 0.4	-	-
*Aeromonas veronii*	-	-	-	-	-
*Chryseobacterium indologenes*	-	-	7.5 ± 0.5	-	-
*Edwardsiella piscicida*	-	21.8 ± 0.6	-	-	-
*Enterococcus faecalis*	-	7.7 ± 0.3	-	-	-
*Lactococcus garvieae*	-	-	-	-	-
*Photobacterium damselae*	9.2 ± 0.4	-	-	-	-
*Plesiomonas shigelloides*	-	22.8 ± 1.6	-	-	-
*Proteus vulgaris*	13.7 ± 0.4	-	-	-	-
*Pseudomonas aeruginosa*	-	-	-	9.4 ± 0.2	-
*Pseudomonas parafulva*	17.5 ± 0.9	-	-	-	-
*Pseudomonas putida*	-	-	-	-	-
*Shewanella xiamenensis*	-	-	-	13.9 ± 0.4	-
*Streptococcus agalactiae*	-	-	-	-	-
*Vibrio alginolyticus*	-	-	14.9 ± 0.8	-	9.2 ± 0.2
*Vibrio cholerae*	-	-	-	-	-
*Vibrio parahaemolyticus*	-	-	-	-	-

Note: data are presented as mean ± SD from three independent biological replicates. For each biological replicate, measurements from three technical replicates were averaged before calculation of the final mean and SD. “-” = no detectable inhibition zone.

**Table 2:** Physiological–biochemical tests of the 5 isolated strains.

Indicators	*P. shigelloides* MS-24	*A. dhakensis* MS-26	*L. lactis* MS-54	*B. subtilis* MS-67	*B. paranthracis* MS-68
MR-VP	−	+	−	+	+
ONPG	+	+	−	+	−
Ur	−	−	−	−	−
GH	−	+	−	+	+
AD	+	+	+	−	+
OD	+	+	−	−	−
LD	+	+	−	−	−
Ox	−	+	+	+	−
0% NaTW	+	+	+	+	+
3% NaTW	+	+	+	+	+
6% NaTW	−	+	−	+	+
8% NaTW	−	+	−	−	+
10% NaTW	−	−	−	−	−
1% NaCl Mal	−	+	−	−	−
1% NaCl Mae	−	+	+	+	−
1% NaCl La	+	+	+	−	−
1% NaCl Ce	−	+	+	−	+
1% NaCl Ar	−	+	−	−	−
1% NaCl Su	−	+	+	+	−
Grow at 42 °C	+	+	−	+	−

Notes: MR-VP: Methyl Red (MR) and Voges–Proskauer (VP); ONPG: o-nitrophenyl-β-D-galactopyranoside; Ur: Urease; GH: Gelatin hydrolysis; AD: Arginine dihydrolase; OD: Ornithine decarboxylase; LD: Lysine decarboxylase; Ox: Oxidase; NaTW: NaCl tryptone water; Mal: Mannitol; Mae: Mannose; La: Lactose; Ce: Cellobiose; Ar: Arabinose; Su: Sucrose; +: Positive; −: Negative.

**Table 3:** Safety assessment of the 5 antagonistic strains on largemouth bass.

Strain Number	Bacterial Species	Mortality (%)
MS-24	*Plesiomonas shigelloides*	67.78 ± 3.85
MS-26	*Aeromonas dhakensis*	96.67 ± 3.33
MS-54	*Lactococcus lactis*	0.00
MS-67	*Bacillus subtilis*	1.11 ± 1.92
MS-68	*Bacillus paranthracis*	81.11 ± 6.94

**Table 4:** The protective effects of *Lactococcus lactis* MS-54 and *Bacillus subtilis* MS-67 on largemouth bass.

Pathogenic Bacteria	Mortality (%)				RPS (%)		
	Control	MS-54	MS-67	MS-54 + MS-67	MS-54	MS-67	MS-54 + MS-67
*Aeromonas salmonicida*	92.22 ± 5.67 ^a^	86.67 ± 5.44 ^a^	ND	85.56 ± 6.29 ^a^	6.02	ND	7.23
*Chryseobacterium indologenes*	43.33 ± 5.44 ^a^	40.00 ± 8.16 ^a^	ND	41.11 ± 6.85 ^a^	7.69	ND	5.13
*Vibrio alginolyticus*	92.22 ± 4.16 ^a^	81.11 ± 6.85 ^b^	ND	80.00 ± 5.44 ^b^	12.05	ND	13.25
*Aeromonas jandaei*	93.33 ± 5.44 ^a^	ND	73.33 ± 7.20 ^b^	72.22 ± 6.28 ^b^	ND	21.43	22.62
*Pseudomonas aeruginosa*	66.67 ± 5.44 ^a^	ND	63.33 ± 7.20 ^a^	62.22 ± 6.85 ^a^	ND	5.00	6.67
*Shewanella xiamenensis*	74.44 ± 6.85 ^a^	ND	66.67 ± 5.44 ^a^	67.78 ± 8.31 ^a^	ND	10.45	8.96

Notes: “ND” = not determined. Different letters indicate significant differences. *p* ˂ 0.05.

## Data Availability

The original contributions presented in this study are included in the article/Supplementary Material. Further inquiries can be directed to the corresponding authors.

## Supplementary Materials

The following supporting information can be downloaded at: https://www.mdpi.com/article/10.3390/microorganisms14102343/s1, Figure S1. Phylogenetic trees of the *16S rRNA* (A) and *gyrB* (B) genes of strain MS-24; Figure S2. Phylogenetic trees of the *16S rRNA* (A) and *gyrB* (B) genes of strain MS-26; Figure S3. Phylogenetic trees of the *16S rRNA* (A) and *gyrB* (B) genes of strain MS-54; Figure S4. Phylogenetic trees of the *16S rRNA* (A) and *gyrB* (B) genes of strain MS-67; Figure S5. Phylogenetic trees of the *16S rRNA* (A) and *gyrB* (B) genes of strain MS-68.
