# Supplementary material for: Screening and Evaluation of Host-Derived Intestinal Probiotic Candidates Lactococcus sp. and Bacillus sp. from Largemouth Bass for Bacterial Disease Biocontrol
Source: Microorganisms. 2026 Oct 14;14(10):2343. doi: 10.3390/microorganisms14102343 (PMC13707846; doi:10.3390/microorganisms14102343)
Supplement: Supplementary file 1 [file microorganisms-14-02343-s001.zip › microorganisms-4568528-supplementary.pdf]

Supplemental Information for

Screening and Evaluation of Host-Derived Intestinal Probiotic Candidates *Lactococcus* sp. and *Bacillus* sp. from Largemouth Bass for Bacterial Disease Biocontrol

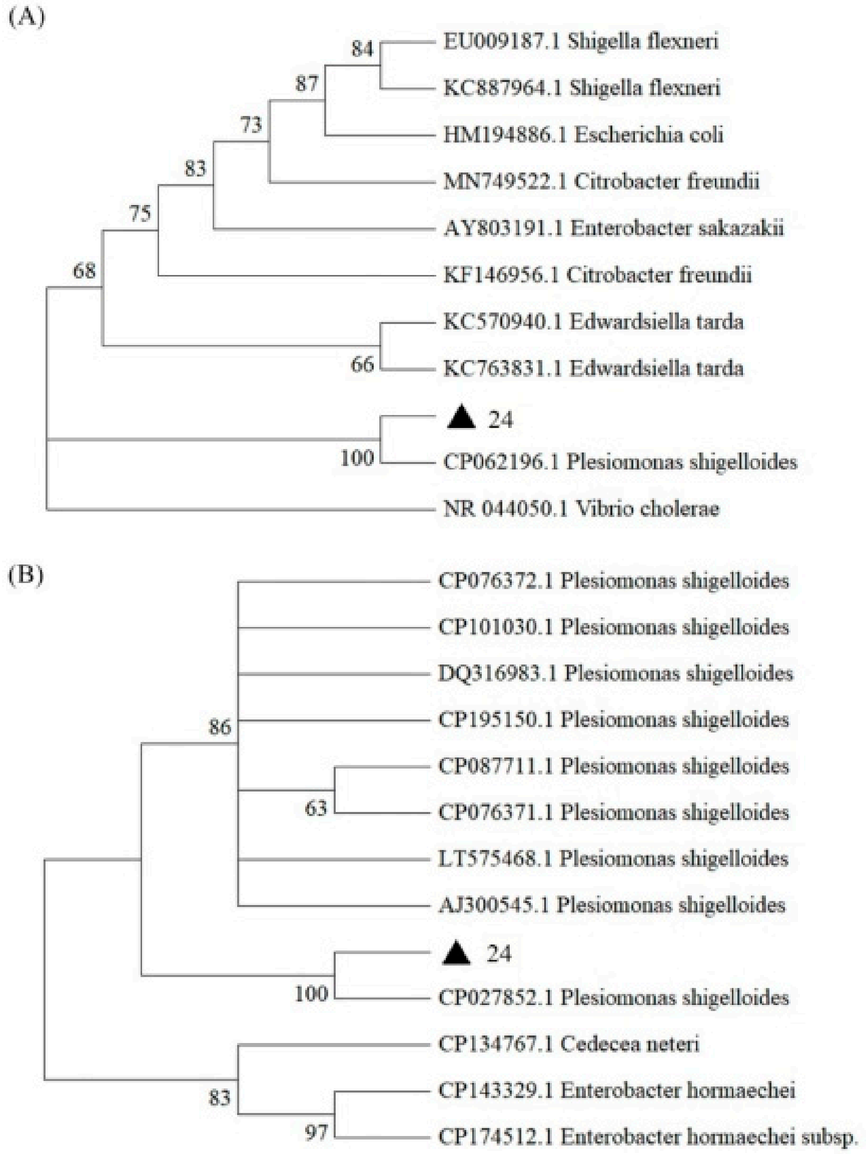

Figure S1. Phylogenetic trees of the *16S rRNA* (A) and *gyrB* (B) genes of strain MS-24.

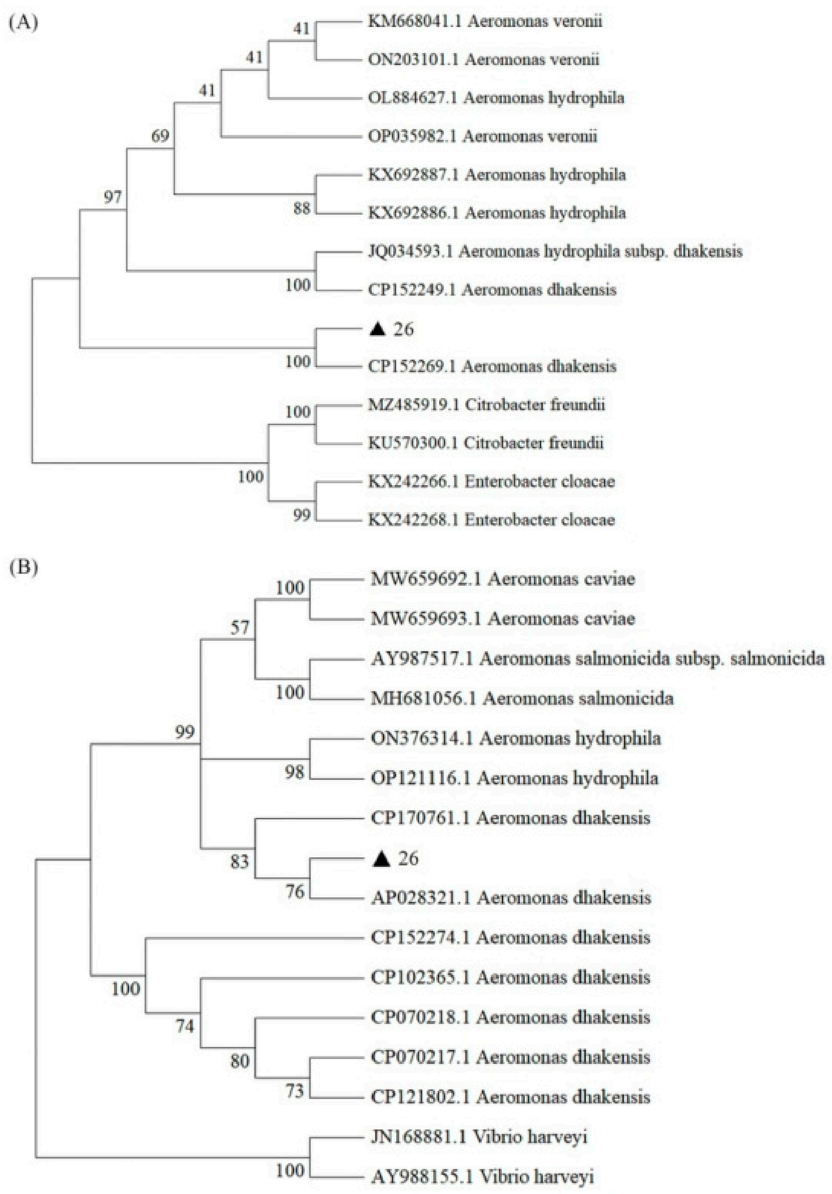

Figure S2. Phylogenetic trees of the *16S rRNA* (A) and *gyrB* (B) genes of strain MS-26.

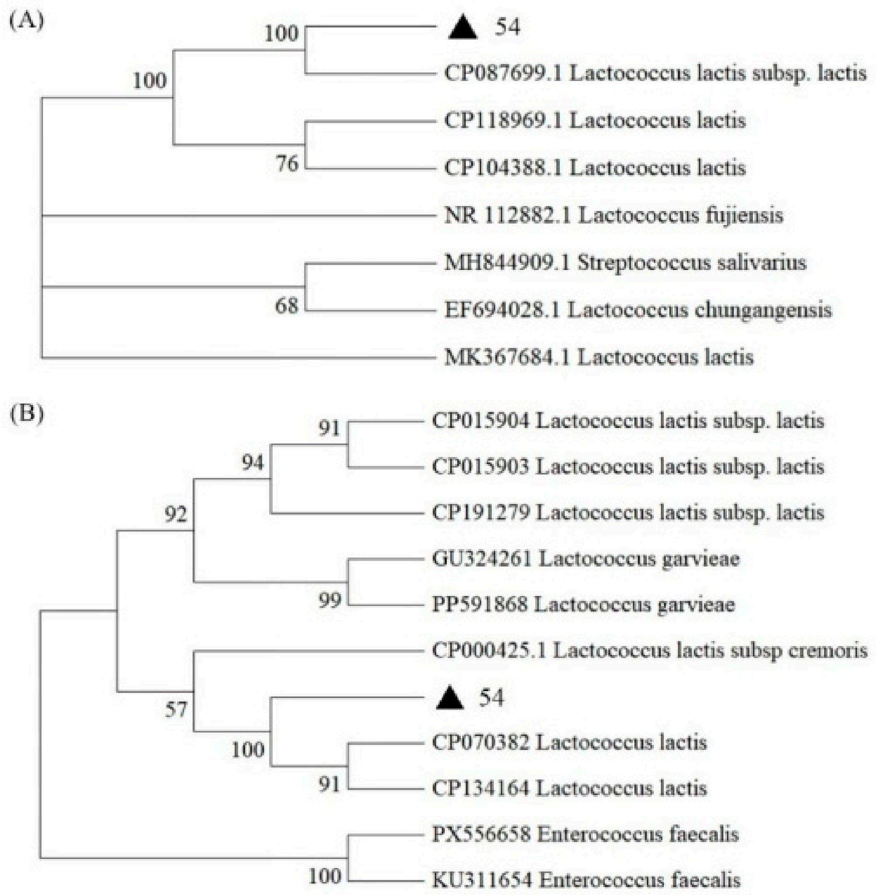

Figure S3. Phylogenetic trees of the *16S rRNA* (A) and *gyrB* (B) genes of strain MS-54.

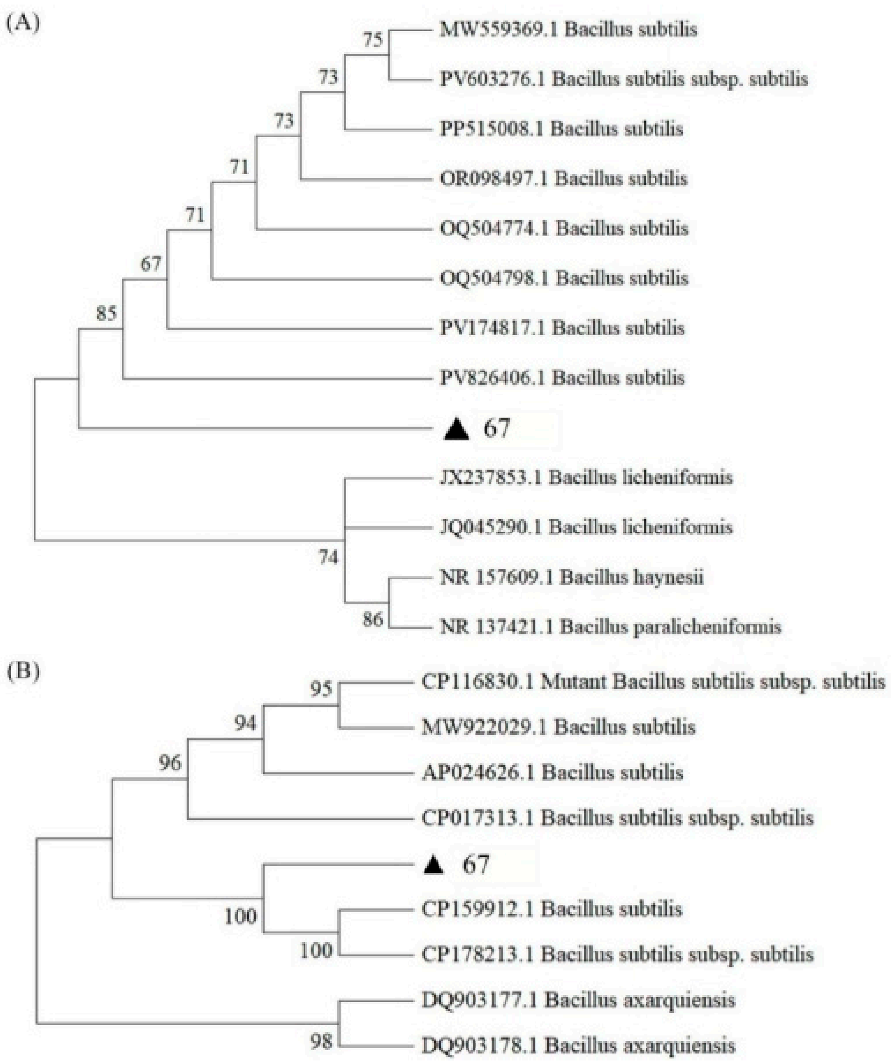

Figure S4. Phylogenetic trees of the *16S rRNA* (A) and *gyrB* (B) genes of strain MS-67.

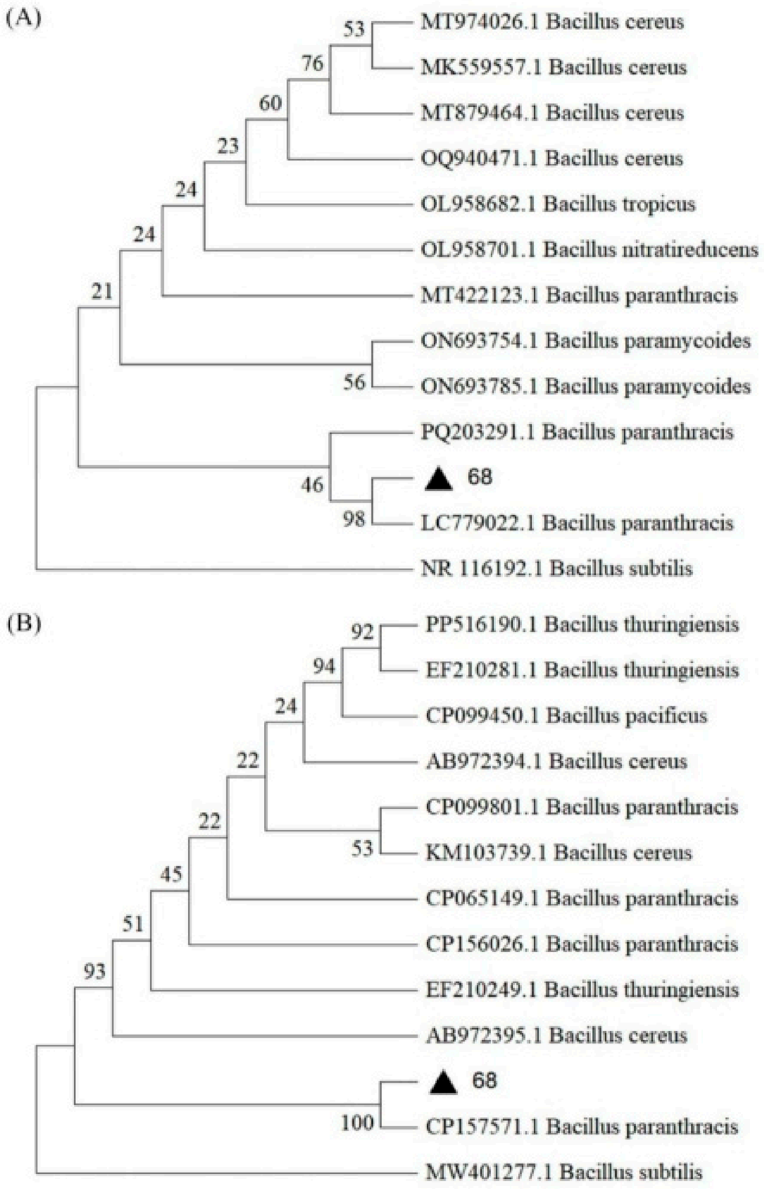

Figure S5. Phylogenetic trees of the *16S rRNA* (A) and *gyrB* (B) genes of strain MS-68.
